# PEGylated Micro/Nanoparticles Based on Biodegradable Poly(Ester Amides): Preparation and Study of the Core–Shell Structure by Synchrotron Radiation-Based FTIR Microspectroscopy and Electron Microscopy

**DOI:** 10.3390/ijms25136999

**Published:** 2024-06-26

**Authors:** Davit Makharadze, Temur Kantaria, Ibraheem Yousef, Luis J. del Valle, Ramaz Katsarava, Jordi Puiggalí

**Affiliations:** 1 Departament de Enginyeria Química, Universitat Politècnica de Catalunya, EEBE, Av. Eduard Maristany 10-14, 08019 Barcelona, Spain; davit.makharadze@upc.edu (D.M.); luis.javier.del.valle@upc.edu (L.J.d.V.); 2 Institute of Chemistry and Molecular Engineering, Agricultural University of Georgia, Tbilisi 0159, Georgia; t.kantaria@agruni.edu.ge (T.K.); r.katsarava@agruni.edu.ge (R.K.); 3 ALBA Synchrotron Light Facility, Carrer de la Llum 2-26, Cerdanyola del Vallès, 08290 Barcelona, Spain; iyousef@cells.es; 4 Barcelona Research Center in Multiscale Science and Engineering, Universitat Politècnica de Catalunya, Campus Diagonal-Besòs, Av. Eduard Maristany 10-14, 08019 Barcelona, Spain

**Keywords:** poly(ester amide), polyethylene glycol, nanoparticles, microspectroscopy, protein adsorption, electron microscopy

## Abstract

Surface modification of drug-loaded particles with polyethylene glycol (PEG) chains is a powerful tool that promotes better transport of therapeutic agents, provides stability, and avoids their detection by the immune system. In this study, we used a new approach to synthesize a biodegradable poly(ester amide) (PEA) and PEGylating surfactant. These were employed to fabricate micro/nanoparticles with a core–shell structure. Nanoparticle (NP)-protein interactions and self-assembling were subsequently studied by synchrotron radiation-based FTIR microspectroscopy (SR-FTIRM) and transmission electron microscopy (TEM) techniques. The core–shell structure was identified using IR absorption bands of characteristic chemical groups. Specifically, the stretching absorption band of the secondary amino group (3300 cm^−1^) allowed us to identify the poly(ester amide) core, while the band at 1105 cm^−1^ (C-O-C vibration) was useful to demonstrate the shell structure based on PEG chains. By integration of absorption bands, a 2D intensity map of the particle was built to show a core–shell structure, which was further supported by TEM images.

## 1. Introduction

The use of Nanoparticles (NPs) has become very active in recent years due to their unique properties and potential applications in various fields of medicine. NPs can be made from multiple materials, such as metals, polymers, lipids, and quantum dots [1,2,3], that offer the ability to modify their properties for specific applications. Typically, drug carrier particles need to meet specific requirements to be effective for various treatments. Stability is one of the most important factors, primarily affected by the zeta potential which plays a pivotal role during drug delivery processes, zeta potential can significantly affect the behavior and performance of particles in terms of association with biological tissues, and therapeutic effectiveness; generally, positively charged NPs exhibit better interaction with the negatively charged cell membrane, hence, they are often used in gene delivery allowing high DNA binding. Biocompatibility is another crucial aspect as particles should not cause harmful side effects and prevent undesirable immune reactions or damage to healthy cells. Finally, drug carrier particles must be capable of providing a sustained release and preferably targeting specific cells or tissues.

Drug encapsulation inside particles is an effective tool for creating delivery systems that offer several advantages over traditional methods. Particles can protect drugs from enzymatic degradation, extend blood circulation time in the body, and increase drug bioavailability. NP drug delivery has shown promising results in the treatment of various diseases, including cardiovascular disease [4], ocular [5,6], and neurological disorders [7]. NPs are actively used as delivery systems for anticancer [8] and anti-inflammatory drugs [9,10]. However, when nanosystems are in a physiological environment, a hard or soft “corona” can be formed as a consequence of the rapid adsorption of biomolecules such as proteins (opsonins) and lipids on their surface [11]; therefore, it can change the size, surface chemistry, solubility, surface charge, and finally, aggregation can take place, and hence biodistribution, cellular uptake, and macrophage capture of NPs [12]. In general, particles coated with hydrophilic polymers show improved circulation properties and decreased macrophage recognition. Various hydrophilic polymers, mostly polyethylene glycol (PEG) and poly(vinyl alcohol), have been studied for drug delivery purposes. Particles decorated with a PEG cloud adsorb less protein from the biological environment and have a longer circulation in the blood [13]; PEG is a biocompatible and non-cytotoxic polymer that is often used to “protect” drug-carrying vehicles and prevent their rapid metabolism. The PEG stealth effect is particularly effective for improving the stability and the circulation time generally required for liposomes [13,14]. Particles coated with heterobifunctional PEG are often used in various targeted drug delivery therapies, these PEG linkers allow chemical modification or conjugation of the distal group employing various antibodies or proteins, which increases the ability of particles to deliver a cytotoxic drug into the target cells [15].

### Stealth Effect and PEGylation Strategies

The stealth effect of PEG and drug carrier protection from opsonins highly depend on the polymer arrangements; hence, PEG can lead to mushroom and brush structures depending on the density of the polymer chains and the interactions with the substrate [16]. It is known that PEG increases the water solubility of encapsulated drugs [17], enhances particle hydrophilicity, and favors cellular internalization [18]. It was shown that PEG-NP accumulation in different organs is independent of the molecular weight of PEG [19]; however, it was demonstrated that PEG molecular weights larger than 2KD are required for an effective stealth effect [20]. In the same way, authors indicate threshold limits in terms of PEG molecular weight, beyond which an increase in molecular weight does not cause a significant change in protein adsorption [21,22].

The coating strategies of particle surfaces with PEG can be accomplished through different routes, including physical adsorption, covalent attachment, or self-assembly techniques. The selection of the most appropriate method depends on factors such as the particle composition, desired stability, and the specific application. Physical adsorption is the simplest approach since it can be attained through the incubation of particles in a PEG solution [23]. Weak interactions are established and consequently, it is expected to have low stability and a potential PEG detachment over time. Covalent attachment entails the chemical coupling of PEG molecules with surface-addressed functional groups present on the particle surface. This could be accomplished by attaching reactive PEG derivatives (i.e., PEG with terminal functional groups like amino and thiol) to reactive sites on the particle surface. Covalent attachment provides powerful and more stable bonding compared to physical attachment. Self-assembly techniques exploit amphiphilic PEG derivatives (e.g., PEG-cholesterol [24], PEG-phospholipids [25,26], or PEG-block copolymers [27] which can quickly assemble on the particle surface and form a stable amphipathic structure. These self-assembled structures can provide enhanced stability and control over the PEG density on the particle surface.

Several techniques are available to study the structure of the particles, such as scanning electron microscopy, atomic force microscopy, and transmission electron microscopy (TEM); however, few of them show the particle structure using synchrotron radiation-based FTIR microspectroscopy (SR-FTIRM). An important advantage of this technique is that it is a non-destructive method and can be used to analyze samples without a complex sample preparation. This method can be particularly useful for studying biological tissues or other complex samples that are difficult to prepare or handle. A synchrotron radiation-based infrared source has approximately a thousand times greater power than the traditional IR source, offering enhanced sample spatial resolution and spectral quality. SR-FTIRM has been successfully used for cancer diagnosis, revealing biological markers of cancer such as the RNA/DNA ratio during carcinogenesis [28]. It was employed for predicting prostate cancer and has a great potential to differentiate localized and aggressive cancer tissues [29]. SR-FTIRM can also be exploited as a powerful tool in the diagnosis of various bacterial and fungal diseases showing priority over traditional methods that require long-term measurements for analysis [30].

In the current study, we investigated and confirmed the core–shell structure of new PEG-coated polymeric particles based on biodegradable polymers using TEM and SR-FTIR. Specifically, for the core of the particle selected a pseudo-protein (PP) biodegradable poly(ester amide) (PEA) labeled as 8L6 and composed of sebacic acid (8), L-leucine (L), and 1,6-hexanediol (6). The polymer was synthesized using an interfacial polycondensation (IP) which is by far a faster and cost-effective method when compared with the solution active polymerization (SAP) performed previously [31]. Moreover, IP results in polymers with higher purity and those that are free of low-molecular-weight organic by-products. One of the main advantages of the amino acid-based PEAs as biodegradable biomaterials over typical polyesters (PE) (such as poly-α-hydroxy acids polyglycolide, polylactide, polycaprolactone, and their copolymers) is the existence of amide groups in the polymer backbone that provides better tissue compatibility since these polymers break down into naturally occurring amino acids; the resulting degradation products are likely to have minimal systemic toxicity. An important feature of polyester degradation is the production of acidic by-products during its hydrolytic breakdown which can trigger an inflammatory response. (e.g., biodegradation of poly-α-hydroxy acids produce large amounts of acidic products like glycolic and lactic acids that have low pK_a_, i.e., 3.83 and 3.86, respectively). Additional advantages of PEAs are synthesis under normal atmospheric conditions via interfacial polycondensation at moderate temperatures (cf.—PEs are synthesized via ring opening polymerization at high temperatures in melt without access to moisture), the wide range of desirable material properties, longer shelf life, etc.

An unsaturated poly(ester amide) derived also from L-leucine to improve compatibility with the core and fumaric acid to allow the attachment of PEG was also synthesized via IP.

## 2. Results

### 2.1. Characterization of Saturated and Unsaturated PEAs

**Scheme 1:**
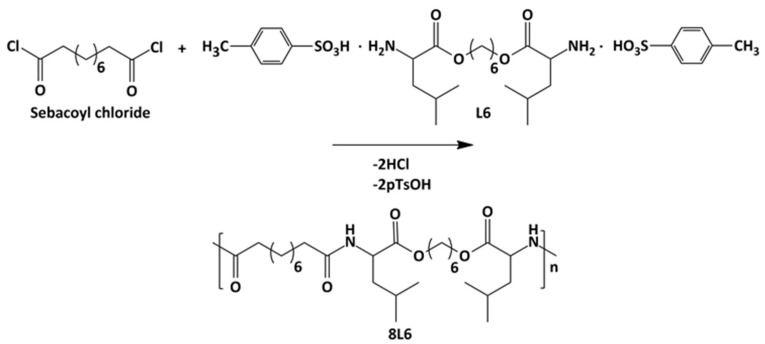
Synthesis of polymer 8L6.

**Figure 1:**
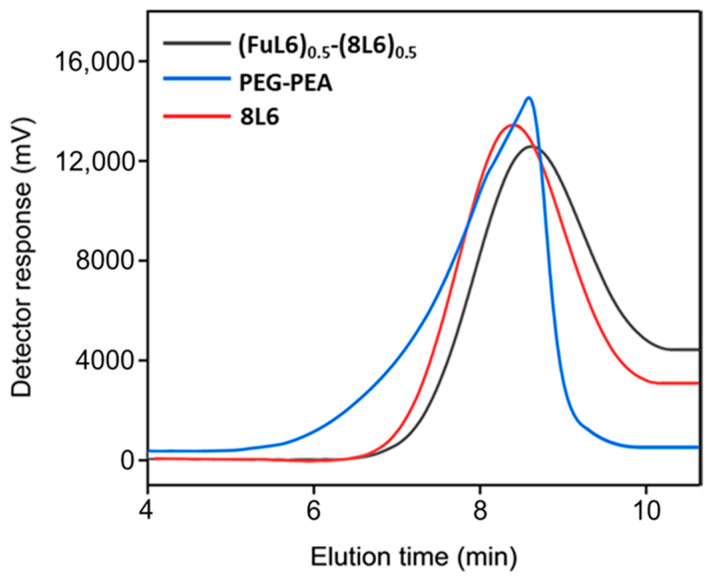
GPC graphs of the synthesized polymers.

Poly(ester amide) 8L6 was obtained with a 90% yield from the polycondensation reaction (Scheme 1) based on the use of the diacyl chloride and L6 monomers; it was synthesized with a higher purity compared with the previously reported synthesis based on the p-nitrophenol active ester of the dicarboxylic acid [31]. Important bis-nucleophilic monomers for building PPs are made of widely accessible, cost-effective materials such as amino acids (AAs), diols, and p-toluenesulfonic acid. The process of monomer preparation as stable di-p-toluenesulfonic acid salts (TDADEs) is quite easy and economical, which includes direct condensation of two moles of α-amino acids with one mole of diols in the presence of two moles of p-toluenesulfonic acid. High yields are achieved during the synthesis of the TDADEs, monomers are purified by recrystallization from water, and show high stability during storage. A weight average molecular weight of 40,500 g/mol and a low polydispersity index (1.72) were determined from GPC analysis (Table 1 and Figure 1); polycondensation gave rise to similar molecular weights using sebacoyl dichloride instead of di-p-nitrophenyl sebacate (M_w_ = 40,500 g/mol). FTIR and NMR spectra (see supplemental information) were fully consistent with the chemical structure. Characteristic FTIR absorption bands of ester (C=O stretching at 1736 cm^−1^), amide (amides A, B, I, and II at 3300, 3080, 1635, and 1538 cm^−1^, respectively), and aliphatic (asymmetric and symmetric CH_2_ stretching and CH_3_ stretching at 2917, 2850 and 2955 cm^−1^, respectively) groups were detected. The broad band centered at 3500 cm^−1^ demonstrated the capability to absorb water due to the presence of the hydrophilic amide groups and also of the terminal amine groups (expected for the slight excess of the L6 monomer in the polycondensation reaction) or even carboxylic acid groups.

^1^H-NMR spectrum of the 8L6 polymer was identified by signals at 0.84–0.98 (12H, -CH_3_), 1.18–1.33 (12H, O-(CH_2_)_2_-(CH_2_)_2_-(CH_2_)_2_-O- and -(O)C-(CH_2_)_2_-(CH_2_)_4_-(CH_2_)_2_-C(O)-), 1.34–1.80 (14H, -CH(CH_3_)_2_, CH_2_-CH(CH_3_)_2_, -O-CH_2_-CH_2_ and -(O)C-CH_2_-CH_2_), 2.16–2.41 (4H, -(O)C-CH_2_), 4.15 (4H, O-CH_2_), 4.66 (2H, NH-CH) and 5.99–6.2 (2H, NH).

**Scheme 2:**
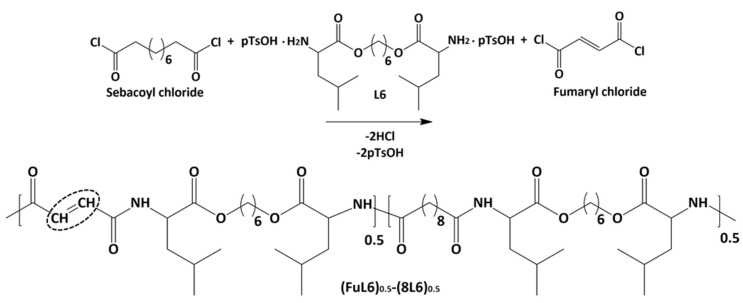
Synthesis of the unsaturated co-poly(ester amide) (FuL6)_0.5_-(8L6)_0.5_. Double bonds are fundamental for attaching hydrophilic PEG molecules.

**Scheme 3:**
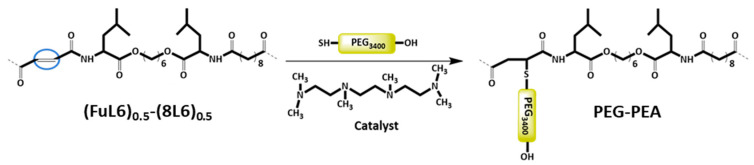
Heterobifunctional PEG attachment to the unsaturated PP–*co*-PEA (FuL6)_0.5_-(8L6)_0.5_ copolymer.

Scheme 2 shows the synthesis of the (FuL6)_0.5_-(8L6)_0.5_ copolymer which was selected to be compatible with the polymer core and as an appropriate precursor to perform the PEGylating surfactant synthesis. This copolymer contained 50/50 (mole/mole) of unsaturated and saturated repeat units and was specially designed to take profit of the capacity of fumaric acid units to covalently attach the heterobifunctional thiol PEG-derivatives using the Michael addition reaction (as depicted in Scheme 3). The presence of saturated (8L6) fragments allows for improving the compatibility between the PEGylated surfactant with the NP core which is based on polymer 8L6 (i.e., to facilitate the anchoring of the surfactant onto the NP surface). The copolymer was obtained with an 85% yield and a moderate molecular weight (M_w_ = 22,200 g/mol, Table 1 and Figure 1) as a consequence of the lower reactivity of the fumaryl dichloride monomer. FTIR spectrum was similar to that displayed by the 8L6 homopolymer; logically, the main difference in the lower intensity of CH_2_ bands was caused by the substitution of 50% sebacate units by fumarate units. Furthermore, a clear increase in the band at 3500 cm^−1^ was detected as evidence of the lower molecular weight and the impact of terminal amine groups.

The ^1^H-NMR spectrum of the copolymer (see supplemental information) showed signals at 0.83–0.91 (12H, -CH_3_), 1.23–1.31 (8H,-O-(CH_2_)_2_-(CH_2_)_2_-(CH_2_)_2_-O- and -(O)C-(CH_2_)_2_-(CH_2_)_4_-(CH_2_)_2_-C(O)-, stack), 1.48–1.62 (12H, -CH(CH_3_)_2_; CH_2_-CH(CH_3_)_2_; -O-CH_2_-CH_2_ and -(O)C-CH_2_-CH_2_, stack), 2.10 (2H, -(O)C-CH_2_), 4.02 (4H, O-CH_2_), 4.25–4.35 (2H, NH-CH), 6.91 (1H, -CH=CH), and 8.10 and 8.77 (2H, NH). The two main NH signals can be observed according to their link with the sebacic acid (8.10 ppm) or the fumaric acid (8.77 ppm) units. Splitting of the NH-CH-CO amino acid proton was also observed according to the neighboring unit (i.e., 4.35 ppm for fumarate and 4.25 ppm sebacate). Note that the split peaks always have a similar area in agreement with the 50% molar composition. The presence of the singlet at 6.91 ppm that indicates the presence of fumarate units and the absence of peaks associated with the corresponding terminal group is remarkable. The copolymer has amine terminal groups as indicated by the two small signals that appear close to the NH signals. Nevertheless, the existence of some carboxylic acid groups associated with the sebacate unit is not discarded. ^13^C-NMR spectra revealed the presence of small a CO signal close to the characteristic one for the sebacate unit.

For surfactant synthesis, heterobifunctional PEG with a molecular weight of 3.4 kDa was used. The molecular weight of PEGylated surfactant could be of great importance in terms of molecular interactions during the self-assembling process because the major forces governing the formation of self-assembled structures are hydrogen bonding, hydrophobic interactions, and Van der Waals forces. In general, increasing the molecular weight of PEG can increase the circulation time of the particle in the bloodstream which is attributed to better steric hindrance from protein adsorption, preventing opsonization [32].

**Figure 2:**
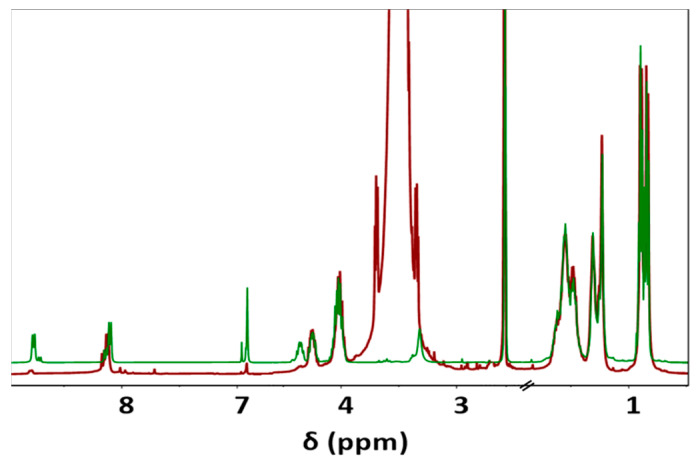
Comparison between ^1^H-NMR spectra of unsaturated co-PEA (FuL6)_0.5_-(8L6)_0.5_ (Green) and PEG-PEA (Red), main signals are observed at 0.83–0.90 (12H, -CH_3_), 1.23–1.31 (8H, -O-(CH_2_)_2_-(CH_2_)_2_-(CH_2_)_2_-O- and -(O)C-(CH_2_)_2_-(CH_2_)_4_-(CH_2_)_2_-C(O)-), 1.48–1.54 (12H, -CH(CH_3_)_2_; CH_2_-CH(CH_3_)_2_; -O-CH_2_-CH_2_ and -(O)C-CH_2_-CH_2_), 2.10 (2H, -(O)C-CH_2_), 3.52 (-O-CH_2_-CH_2_-O- of PEG), 4.02 (4H, O-CH_2_), 4.23–4.32 (2H, NH-CH), 6.91 (-CH=CH-), and 8.11 and 8.76 (2H, NH).

During the Thiol addition reaction, electron-deficient alkene is attacked by the thiol group nucleophilically, which leads to the creation of a carbon–sulfur bond [33,34,35,36] and an increase in the final molecular weight; the reaction is often employed to enhance the pharmacokinetic characteristics of different compounds, such as proteins, by the covalent link with PEG chains. The weight average molecular weight of the unsaturated copolymer before the PEG attachment was 22,200 g/mol, after the addition reaction, it significantly increased (i.e., up to 39,900 g/mol) as summarized in Table 1 and shown in Figure 1, indicating a successful PEG attachment. The signal intensity of hydrogen atoms adjacent to a carbon–carbon double bond drastically decreased (Figure 2), meanwhile, the signal intensity of hydrogen atoms of the CH_2_ group of the polyethylene glycol (3.52 ppm) chain increased. Consequently, a degree of PEGylation (PEG%) was evaluated considering the intensity of the signal at 6.91 ppm (i.e., the CH=CH protons) and a reference signal (e.g., the peak at 0.83–0.91 ppm corresponding to the CH_3_ protons). Applying Equation (1) to the intensity data indicated that a degree of PEGylation close to 90% was attained.

PEG% = (1 – 12 × I_6.91_/I_0.83–0.91_) × 100 (1)

### 2.2. Thermal Properties of the Synthesized Samples

**Figure 3:**
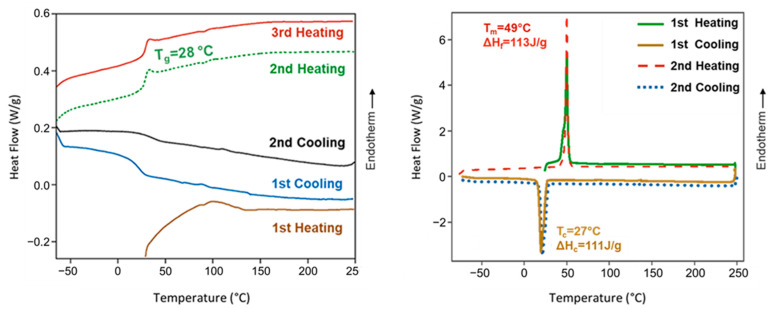
DSC heating and cooling curves for poly(ester amide) 8L6 (**left**) and PEG-PEA (**right**). DSC traces for PEA showed a glass transition temperature, as expected from an amorphous polymer, except the first heating run where interference with adsorbed water predominated. Melting and crystallization peaks of the PEG moieties were only detected in the PEGylated copolymer.

Poly(ester amide) 8L6 was amorphous despite the regular disposition of its constitutive units, the presence of long polyethylene segments (i.e., sebacate), and groups that are able to establish strong intermolecular interactions (i.e., hydrogen bonds for amide and dipolar for the ester groups). Therefore, the large size of the L-leucine residues seems to prevent crystallization. Figure 3 shows that the polymer recovered from the synthesis of an endothermic bump at a temperature close to 100 °C that is attributed to adsorbed water. A change in the baseline corresponding to the glass transition could only be observed in the cooling run after heating the sample to 225 °C and the subsequent heating runs. A minor difference was detected between the temperature at which the sample became brittle on cooling (25 °C) and soft on heating (28 °C). Note that the glass transition temperature is relatively high as a consequence of the strong interactions between amide groups and even due to the presence of the leucine lateral groups. Complete reproducibility was observed between the second and third heating runs, suggesting a high thermal stability. Finally, it should be indicated that on heating, an enthalpy relaxation peak is observed after the glass transition as a consequence of the release of energy after the material has been aged below the glass transition process. In the case of the PEGylated copolymer, the main interesting point is the observation of a melting peak associated with the PEG chains that can crystallize.

**Figure 4:**
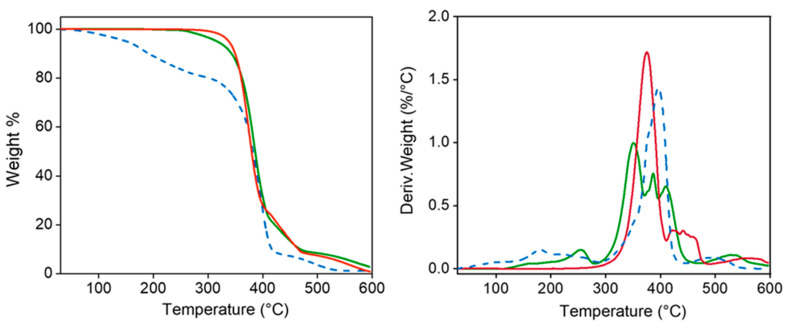
TGA (**left**) and DTGA (**right**) curves for 8L6 (red) and PEG-PEA (dashed blue) polymers and the nanoparticles based on 8L6 prepared with PEG-PEA (green).

TGA and DTGA demonstrated the high thermal stability of the polymers which is essential for evaluating their suitability for sterilization processes (Figure 4). Specifically, decomposition started at a temperature higher than 300 °C, followed by a main single step up to a weight loss of 25% (approximately at 400 °C) and then different minor events. A char yield of 0% was observed at 600 °C. Decomposition of the PEG-PEA sample shows an initial weight loss (20%) between 100 °C and 200 °C, which seems to be associated with adsorbed water. Decomposition between 20% and 90% weight loss is rather complicated since a clear peak and two shoulders can be distinguished in the DTGA curve. The low-temperature shoulder seems to be related to the presence of less stable fumarate units, the second shoulder appears at the same temperature as the peak detected for the PEA, and finally, the high-temperature peak (410 °C) corresponds to the PEG decomposition. A minor decomposition near 500 °C is observed before attaining a weight loss of 100%. Degradation of the particles is more complex but the explained events can still be observed and even DTGA peaks instead of shoulders can be observed.

### 2.3. Nanoparticle Size

**Figure 5:**
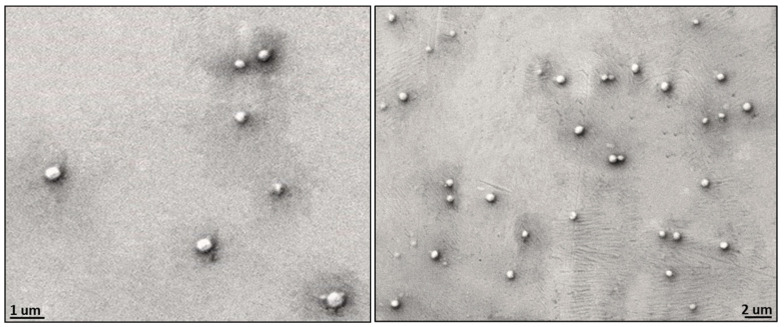
Scanning electron micrographs showing size distribution and morphology of the particles based on polymer 8L6 and PEG-PEA surfactant.

In general, the term “nanoparticle” refers to a particle whose size varies up to 100 nanometers [37] although polymeric NPs are also referred to those size ranges between 1 nm and 1000 nm. Depending on this NP’s size, distribution routes in the human body differ from each other; NPs, especially those larger than 200 nm, tend to accumulate in the spleen due to splenic sinusoids acting as a filter for blood-borne particles [38], whereas NPs less than 5.5 nm have been shown to undergo renal excretion [39]. NP size is also an important parameter in the enhanced permeability and retention phenomena of cancer cells. NPs in the size range of 100–200 [40] nanometers reveal various advantages using the EPR effect; they can passively accumulate in tumor tissues, leading to high concentrations at the tumor site compared to healthy tissues. Particles prepared by the nanoprecipitation method based on PEG-PEA exhibit a rounded morphology and a diameter distribution that varies between 300 nm and 650 nm (Figure 5). PEGylated particles by the DLS method show a relatively narrow size distribution (Table 2); the average size of the PEG-PEA-based 8L6 particles is 175 nm.

### 2.4. Protein Interactions with Nanoparticle Surfaces

**Figure 6:**
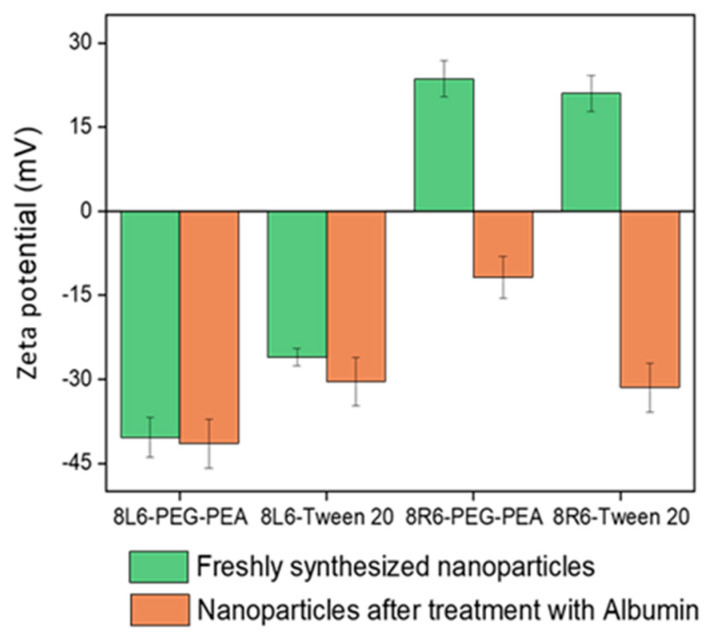
Zeta potential of 8L6 and 8R6 nanoparticles derived from PEG-PEA and tween 20 surfactants before and after treatment with serum albumin.

**Figure 7:**
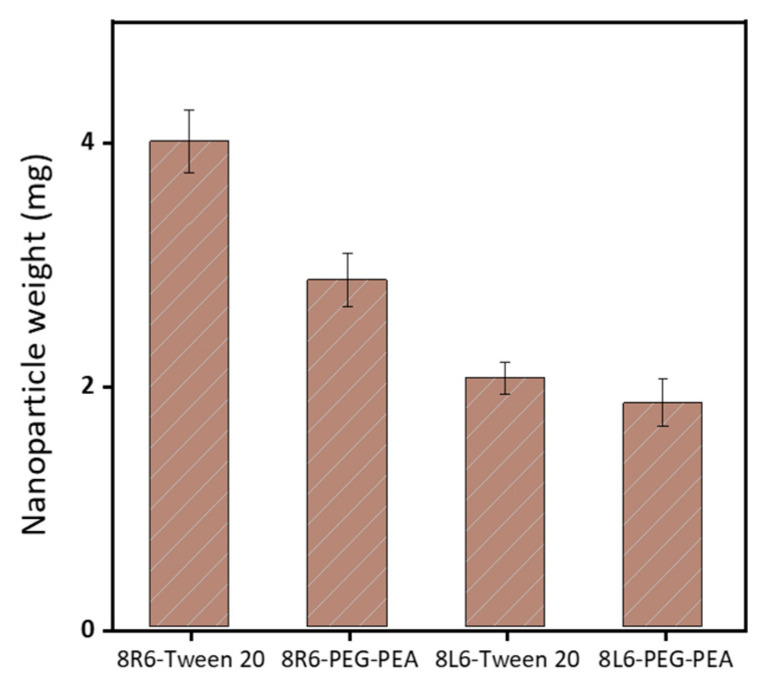
The amount of HSA protein adsorbed to the surface of the four types of considered nanoparticles.

Human serum albumin (HSA) was employed as a model to evaluate how the prepared NPs will interact with different proteins once in the bloodstream. Specifically, the primary interest lies in understanding how NP zeta potential, PEG-PEA, and a conventional surfactant Tween 20 can influence protein adsorption since PEG is known for its “stealth effect” or ability to reduce protein adsorption and increase biocompatibility. This is a crucial point because adhered blood proteins enhance macrophage recognition and finally clearance from the body. At neutral pH, HSA is negatively charged (Figure 6) and consequently, the zeta potential change is insignificant among nanoparticles made of PP 8L6, while nanoparticles with positive zeta potential closely interact with the negatively charged HSA. In the case of 8R6-PEG-PEA, the zeta potential changed from +23.14 mV to −12.06 mV, and in the case of 8R6-Tween 20, from +19.6 mV to −30.42 mV. The essential difference between NP zeta potential after treatment with albumin could be attributed to the higher protective effect of the PEG cloud (in comparison with the surfactant tween 20). Across the four samples, the smallest change in zeta potential was observed for 8L6 particles protected with the PEG cloud. Figure 7 shows that the protein adsorption is the highest in the case of 8R6-Tween 20 followed by 8R6-PEG-PEA > 8L6-Tween 20 > 8L6-PEG-PEA; therefore, the charge of the particle has the greatest influence on its interaction with the HSA protein, although a minor impact of the stealth effect of PEG should also be considered. In summary, 8L6 appears as a good candidate for minimizing the adsorption of the most abundant proteins in the bloodstream, also being positive for the effect caused by the existence of a PEG corona.

### 2.5. Synchrotron Radiation-Based Infrared Microspectroscopy and TEM Investigations

**Figure 8:**
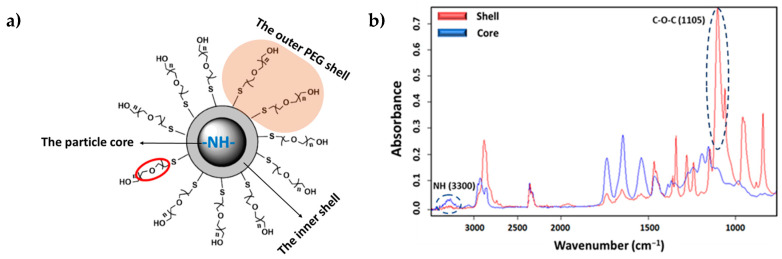
(**a**) Core–shell structure of a typical PEG-PEA particle with an indication of the localization of characteristic chemical groups of PEG and PEA. (**b**) Comparison between absorbance infrared spectra of the particle core (blue) and shell (red) with labeling of the bands of the selected representative groups.

The ST-FTIR measurement was performed using a 36× Schwarzschild objective having a numerical aperture of 0.52. However, the reduction of the transmitted infrared radiation (due to the diffraction limit of confining the SR beam size below 10 × 10 µm^2^) may cause noise limitations in the FTIR measurements. Therefore, analysis was carried out with micro-sized (7 μm) PEGylated particles to clearly distinguish the differences in chemical composition. Two specific IR bands were selected for generating the IR chemical images after raster scanning of the sample under the IR microscope mapping (i.e., to generate bidimensional intensity maps from each selected band). Specifically, the C-O-C anti-symmetric stretching (1105 cm^−1^) and the amide I (3300 cm^−1^) bands (Figure 8b) are characteristics of PEG (shell) and PEA (core); therefore, they were chosen to reveal the different chemical compositions of the shell and the core of the particle.

**Figure 9:**
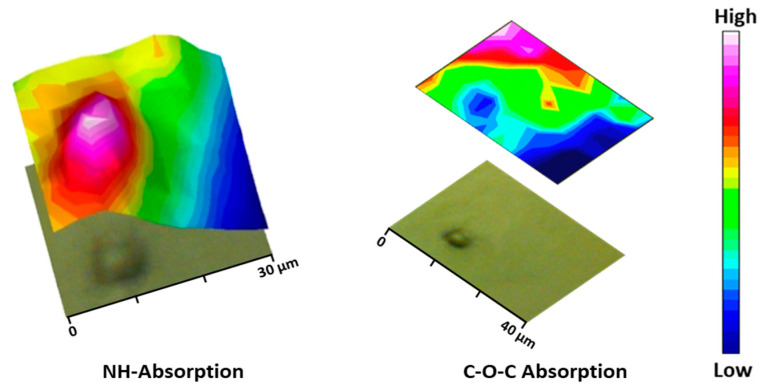
Micrographs of the PEGylated microparticles’ corresponding bi-dimensional intensity maps were obtained by integration of the NH (**left**) and C-O-C (**right**) bands.

During the particle preparation, the self-assembling process occurs spontaneously, the hydrophilic PEG fragments of PEG-PEA chains are oriented outward, interacting with the surrounding aqueous phase to form the particle’s surface; meanwhile, the hydrophobic PEA segments constituted the particle core that separates from the aqueous environment; this structural organization is illustrated in Figure 8a, depicting the self-assembled structure in detail. Note that the unsaturated copolymer surrounds the core forming a corona (gray color). The main difference in FTIR spectra is observed at a 1105 cm^−^*^1^* wavenumber corresponding to the C-O-C anti-symmetric stretching vibration of the PEG chain. The particle core is made of polymer 8L6, containing an NH absorption band. By integrating these two fingerprint peaks, 2D intensity maps were built up (Figure 9), giving information about the chemical group distribution in the particle’s chemical structure. The intensity of the NH band was stronger in the center of the particle and progressively decreased when moved away. On the contrary, the intensity of the C-O-C band was extremely low in the core of the particle and increased significantly in the outer part of the particle.

**Figure 10:**
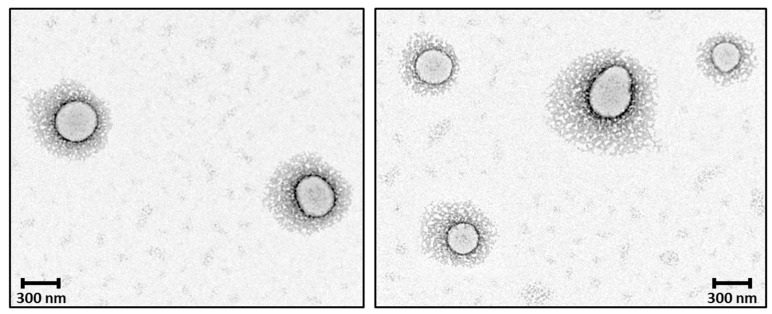
TEM micrographs showing the core–shell structure of the newly prepared nanoparticles; PEG chains have a high coverage density around the nanoparticle core.

The observed morphology of solid 8L6-based particles (Figure 10) confirms the validity of SR-FTIRM results. As can be seen from Graph 10, PEG chains have a high coverage density around the nanoparticle core. The staining solution mainly localizes at the interface between the PEA core and the PEG shell, potentially binding to the amide hydrogen atoms by H-bonding [41] or to the terminal amine or even carboxylic acid groups found in the polymer. Although similar functional groups are presented in both the core and the interface, the interface probably offers a unique environment due to a higher concentration of accessible functional groups, increased surface area, and greater molecular mobility. These factors could create an optimal region for uranyl acetate binding, resulting in enhanced contrast at the core–shell boundary.

## 3. Materials and Methods

### 3.1. Materials

Thiol-PEG-Hydroxy (3.4 kDa) was purchased from Biopharma PEG Scientific, Inc. (Watertown, MA, USA). Protein Assay Kit II (Bio-Rad, Hercules, CA, USA). Sebacoyl chloride (99%), fumaryl chloride (95%), benzoyl chloride (99%), anhydrous diethyl ether (≥99.7%), dimethyl acetamide (DMA) (≥99%), dimethyl sulfoxide (DMSO) (≥99.7%) amylene-stabilized anhydrous dichloromethane (DCM) (≥99.8%), 1,1,4,7,10,10-hexamethyltriethylenetetramine (HMTTA) (97%), sodium carbonate anhydrous powder (99.99%) and the dialysis bag (MWCO 5 kDa), and human serum albumin (≥96%) were purchased from Sigma-Aldrich (Munich, Germany). All the chemicals were used as received.

### 3.2. Methodology

#### 3.2.1. Synthesis of the Core–Shell Particles

Three synthesis steps can be considered: (a) synthesis of a saturated poly(ester amide) rich in the hydrophobic L-leucine amino acid that will be used for the core of the new particles, (b) synthesis of an unsaturated co-poly(esteramide) based on the reaction of the L6 monomer and the appropriated mixture of sebacoyl and fumaryl chloride monomers; this copolymer was subsequently used for the preparation of the shell of new particles (c) PEGylation of the co-poly(ester amide) through reaction with the unsaturated bonds.

#### 3.2.2. Synthesis of the Biodegradable Polymer 8L6

The pseudo-protein 8L6 was used as the basic polymer for the particle preparation. The polymer was synthesized according to Scheme 1 by interfacial polycondensation of sebacoyl chloride with the di-*p*-toluenesulfonic acid salt of the bis(L-leucine)-1,6-hexylene diester (L6) monomer prepared as previously reported (see Ref. [42] and Refs. cited therein). Briefly, the bis-electrophilic monomer L6 (0.065 moles), sodium carbonate (0.163 moles), and distilled water (400 mL) were mixed in a three-necked flask equipped with an overhead stirrer and condenser. The mixture was stirred at room temperature for 40 min until complete dissolution of compounds. Sebacoyl chloride (0.064 M), and amylene-stabilized DCM (200 mL) were put in an Erlenmeyer flask and magnetically stirred at ambient temperature until a homogeneous solution was obtained. The sebacoyl chloride solution was moved to a dropping funnel attached to a three-necked flask and subsequently added dropwise to the first solution vigorously stirred at 1700 rpm. After stirring for 40 additional min, the aqueous (upper) layer was removed mechanically by a syringe. The DCM polymer solution was repetitively washed (three times) with fresh water. Then, 1 L of distilled water was added each time to the flask containing the DCM polymer solution. After stirring the mixture at 200 rpm for 30 min, the system was allowed to stand until the two-phase system was formed and the aqueous solution could subsequently be removed. Finally, DCM was evaporated, and the obtained polymer was vacuum-dried for 24 h at 50 °C (Scheme 1).

#### 3.2.3. Synthesis of the Unsaturated Biodegradable Co-Poly(Ester Amide)

The copolymer was obtained by reaction of the same ratio of fumaryl and sebacoyl chlorides with the stoichiometric amount of the previously synthesized L6 monomer. This copolymer, abbreviated as (FuL6)_0.5_-(8L6)_0.5_ (Scheme 2), was used for the subsequent Michael attachment of the PEGylating surfactant. Briefly, the IP of a sebacoyl chloride (0.032M) and fumaryl chloride (0.032M) mixture with the L6 monomer (0.0653M) was performed according to the above-described steps for the 8L6 poly(ester amide). The unsaturated fumaric acid-based pseudo protein is susceptible to experiment cross-linking reactions involving the terminal amino groups and the activated double bonds of the polymeric backbone. This undesirable crosslinking was especially intensive in a solid state, namely, when the unsaturated polymer was separated from the reaction solution. Hence, to avoid crosslinking during storage, the terminal amino groups of the resulting polymer were capped prior to its separation from the reaction solution. For this, 3.0 g of benzoyl chloride was added to the reaction mixture, which was subsequently stirred for 12 h. The reaction solution was washed in a separating funnel and the (FuL6)_0.5_-(8L6)_0.5_ copolymer was purified and vacuum dried (24 h at 50 °C) as described above for the 8L6 homopolymer.

#### 3.2.4. Thiol-Michael Addition of Heterobifunctional PEG to Unsaturated Co-Poly(Ester Amide)

The thiol–ene addition reaction of the heterobifunctional SH-PEG-OH to unsaturated biodegradable co-poly(ester amide) was base-catalyzed with HMTTA catalyst (Scheme 3); the unsaturated polymer and PEG were obtained in a 1:1 molar ratio (double bond amount/PEG). Specifically, 1.0 g (0.00029 mol) of SH-PEG-OH and 137 mg of (FuL6)_0.5_-(8L6)_0.5_ were charged in a round bottom three-necked flask and dissolved completely in 10 mL of DMA. The flask was equipped with a pipe for constant nitrogen flow and a condenser. HMTTA was injected into the flask and the reaction was performed at 120 °C for 48 h. The completion of the PEGylation reaction was periodically monitored by removing droplets from the reaction medium that were introduced into distilled water. The absence of precipitation indicated a satisfactory degree of PEGylation since water-soluble surfactant molecules were expected in contrast with the initially non-soluble polymer molecules. Subsequently, the reaction mixture was precipitated in 20 mL of diethyl ether to remove the residual HMTTA and finally, the precipitated PEG-PEA polymer was redissolved in a fresh aliquot of 10 mL of DMA and reprecipitated in 20 mL of diethyl ether. The purification process was performed three times in total. The precipitated polymer was dissolved in distilled water, filtered, dialyzed (MWCO 5 kDa) to remove the unbound PEG, and then freeze-dried. ^1^H-NMR was used to check the composition and PEG-attachment efficiency of the resulting grafted PEG-PEA copolymer.

#### 3.2.5. Preparation of Nanoparticles via Precipitation Method

The pseudo-protein 8L6 as a representative of the family of amino acid-based biodegradable polymers was used as a key polymer for the particle preparation. Particles based on an 8L6 core were prepared according to the nanoprecipitation method [43] that was previously used for the preparation of PP-based NPs [44]. To obtain a hydrophilic shell, PEG-PEA was used as a surfactant. Specifically, 30 mg of 8L6 was dissolved in 1.0 mL dimethyl sulfoxide and the obtained solution (organic phase) was added dropwise to 10 mL of water containing 50 mg of the PEG-PEA surfactant (or Tween 20) at a stirring rate of 700 rpm. The suspensions were additionally stirred for 10 min and dialyzed against water to remove DMSO and then were stored at 4 °C in the fridge. 8R6 polymeric NPs were prepared similarly, 30 mg of polymer was dissolved in 1.0 mL of DMSO and precipitated into 10 mL water either containing 50 mg of PEG-PEA or Tween 20. The microparticles for synchrotron-based FTIR microspectroscopy were specially synthesized by modified conditions; specifically, 30 mg of the polymer 8L6 was dissolved in 0.5 mL of DMSO and was added dropwise to 10 mL of water containing 30 mg of the PEG-PEA surfactant at a stirring rate of 700 rpm.

### 3.3. Characterization Techniques

#### 3.3.1. Polymer Characterization

^1^H- and ^13^C-NMR spectra were recorded using a Brucker NMR Ascend spectrometer operating at a magnetic field strength of 400 and 100 MHz, respectively. Approximately, 10 mg of the corresponding sample was dissolved in deuterated dimethyl sulfoxide. Tetramethylsilane (TMS) was used as an internal reference. The spectra were analyzed using the Bruker TopSpin software (version 4.3.0).

The chemical structures of the synthesized polymers were also characterized with FTIR spectroscopy using JASCO 4700 equipment (Jasco Co., Tokyo, Japan). The spectra were recorded in ATR mode by 64 scans and a resolution of 4 cm^−1^ in the range of 4000–600 cm^−1^. The FTIR spectra were processed using the Spectra Manager™ software (Version 2).

The weight average (*M_w_*) and number average (*M_n_*) molecular weights were determined at 38 °C by GPC using a Shimadzu HPLC-420 instrument equipped with a Shimadzu RID-20A differential refractive index detector. GPC analysis was carried out using solvent 1,1,1,3,3,3-hexafluoro-2-propanol (HFIP) containing CF_3_COONa (0.05 M). The flow rate and injected volume were 1.0 mL/min and 20 µL, respectively. GPC columns were calibrated with poly(methyl methacrylate) standards (*M_n_* = 600–1,600,000 g mol^−1^). A polymer sample solution with a concentration of 5 mg/mL was prepared and filtered with PTFE filters (200 nm) prior to injection. The spectra were obtained and processed using Shimadzu’s LabSolutions software (Version 6.50, Shimadzu Co., Kyoto, Japan).

#### 3.3.2. Nanoparticle Size and Morphology

The average hydrodynamic diameter of the particle was measured at 25 °C by dynamic light scattering employing Nanobrook (90 plus) equipment. A few drops of freshly prepared particles were diluted in 1 mL of water and three complete analyses were performed for each sample. Individual measurements consisted of 10 recordings, each of them requiring 30 s. The result is reported as the mean diameter ± SD (standard deviation). In addition, the particle size and morphology were evaluated with scanning electron microscopy (Neon 40, Zeiss, Munich, Germany).

Samples for TEM observations were stained with drops of UranyLess contrast solution deposited on the surface of carbon-coated grids where a drop of diluted suspension of NPs (30 mg/mL) was placed. After incubation for 1 min, the excess fluid was removed and the grid surface was air-dried for 24 h at room temperature before loading into the microscope. High-resolution TEM pictures were obtained by a FEI Tecnai 10 electron microscope, operating at 100 kV.

#### 3.3.3. Investigation of PEGylated Polymeric Microparticles through SR-FTIRM Analysis

Aliquots of the microparticle suspension were diluted 50 times with MilQ water and then the diluted drops were deposited on calcium fluoride discs and left to dry overnight. SR-FTIRM spectra were recorded at the MIRAS beamline at ALBA synchrotron (Cerdanyola del Vallès, Spain) using a Hyperion 3000 Microscope equipped with an HgCdTe (MCT) detector (BRUKER, Ettlingen, Germany) match with 36× magnification condenser having a numerical aperture 0.52, coupled to a Vertex-70 spectrometer. The background spectrum was recorded in a sample-free region using 256 scans to remove any residual contamination surrounding the sample. The measuring range was between 750 and 3500 cm^−1^. The spectra were collected in a transmission mode at 4 cm^−1^ spectral resolution using a 10 × 10 μm^2^ aperture dimension. FTIR data were analyzed using the OPUS software (Version 8.0, Bruker Optics, Ettlingen, Germany).

### 3.4. Nanoparticle Interaction with Human Serum Albumin

Coverage of the NP surface by PEG molecules, or in other terms, the PEG density, determines NP fate. When the surface of the nanoparticle is tightly covered by PEG molecules, it prevents the interaction of blood proteins with the nanoparticle and facilitates the sequestration of the nanoparticle by the reticuloendothelial system. To study how the zeta potential and the PEG-covering surface influenced nanoparticle–protein interaction, a comparative analysis was performed considering four different types of nanoparticles. These were prepared using two different PP polymers: 8R6 based on the amino acid arginine [45], having a positive charge, and 8L6 having a negative zeta potential. In addition, two different surfactants were used for NP stabilization, biodegradable PEGylating surfactant (HO-PEG-PEA) synthesized by our group, and Tween 20. Briefly, 2 mL of NP suspension containing 6 mg of nanoparticles and 2 mL of HSA solution (30 mg/mL) was magnetically stirred for 2 h at neutral pH. Particles were separated by centrifugation at 15,000 rpm subsequently washed with water and centrifuged again. The washing process was repeated three times in total. The amount of HSA was calculated by the Bradford total protein assay, which is based on the shift of the absorbance maximum of Coomassie Brilliant Blue G-250 dye (i.e., from 465 to 595 nm), this assay was carried out according to the protocol provided by the manufacturer (Bio-Rad): 160 μL of each standard and sample solution were put into 96 well plates and 40 μL of dye reagent were added and mixed using a multi-channel pipette. Solutions were subsequently incubated for half an hour isolated from the light. Finally, absorbance was measured at 595 nm using an EZ Read 400 microplate reader (Biochrom Ltd., Cambridge, UK).

## 4. Conclusions

The PEG stealth effect has been widely studied and applied in the field of drug delivery for biological applications and to date, numerous articles are devoted to the study and use of PEGylated particles. We have synthesized a biodegradable amphiphilic polymer based on PEG and a PEA having leucine amino acid units. PEG efficiently performs the role of a stabilizer and surrounds the particle, creating a core–shell structure and making a stealth effect, which is a very important feature for successful drug delivery. In our research, we studied how the PEG cloud and zeta potential of NPs influenced NP–protein interaction, we characterized the core–shell structure using SR-FTIRM coupled with mapping capabilities. In this way, chemical components could be well distinguished inside particles by mapping the sample through the intensity of representative bands. In addition, TEM images of PEG-coated particles provided beneficial information about nanoparticle size, shape, and morphology, that reinforced the SR-FTIRM research findings.

## Figures and Tables

**Table 1:** Molecular weights of the synthesized poly(ester amide)s.

Polymer	*M_w_* (g/mol)	*M_n_* (g/mol)	PDI
8L6	40,500	23,500	1.72
(FuL6)_0.5_-(8L6)_0.5_	22,200	12,200	1.82
PEG-PEA	39,900	20,100	1.99

**Table 2:** Nanoparticle size distribution.

Sample	Particle AD [PDI ± SD]
8L6-PEG-PEA	175 nm (0.121 ± 0.041)
8R6-PEG-PEA	190 nm (0.255 ± 0.021)
8L6-Tween 20	150 nm (0.175 ± 0.033)
8R6-Tween 20	197 nm (0.185 ± 0.015)

## Data Availability

Data is contained within the article and Supplementary Materials.

## Supplementary Materials

The following supporting information can be downloaded at: https://www.mdpi.com/article/10.3390/ijms25136999/s1.

## References

[1] Azizi M., Ghourchian H., Yazdian F., Bagherifam S., Bekhradnia S., Nyström B. (2017). Anti-cancerous effect of albumin coated silver nanoparticles on MDA-MB 231 human breast cancer cell line. Sci. Rep..

[2] Patra C.R., Bhattacharya R., Mukhopadhyay D., Mukherjee P. (2010). Fabrication of gold nanoparticles for targeted therapy in pancreatic cancer. Adv. Drug Deliv. Rev..

[3] Cheng J., Teply B.A., Sherifi I., Sung J., Luther G., Gu F.X., Levynissenbaum E., Radovicmoreno A., Langer R., Farokhzad O.C. (2007). Formulation of functionalized PLGA–PEG nanoparticles for in vivo targeted drug delivery. Biomaterials.

[4] Chan J.M., Rhee J.-W., Drum C.L., Bronson R.T., Golomb G., Langer R., Farokhzad O.C. (2011). In vivo prevention of arterial restenosis with paclitaxel-encapsulated targeted lipid–polymeric nanoparticles. Proc. Natl. Acad. Sci. USA.

[5] Musumeci T., Bucolo C., Carbone C., Pignatello R., Drago F., Puglisi G. (2013). Polymeric nanoparticles augment the ocular hypotensive effect of melatonin in rabbits. Int. J. Pharm..

[6] Yang H., Tyagi P., Kadam R.S., Holden C.A., Kompella U.B. (2012). Hybrid Dendrimer Hydrogel/PLGA Nanoparticle Platform Sustains Drug Delivery for One Week and Antiglaucoma Effects for Four Days Following One-Time Topical Administration. ACS Nano.

[7] Karthivashan G., Ganesan P., Park S.-Y., Kim J.-S., Choi D.-K. (2018). Therapeutic strategies and nano-drug delivery applications in management of ageing Alzheimer’s disease. Drug Deliv..

[8] Wang M., Thanou M. (2010). Targeting nanoparticles to cancer. Pharmacol. Res..

[9] Hirst S.M., Karakoti A.S., Tyler R.D., Sriranganathan N., Seal S., Reilly C.M. (2009). Anti-inflammatory Properties of Cerium Oxide Nanoparticles. Small.

[10] Whitmire R.E., Wilson D.S., Singh A., Levenston M.E., Murthy N., García A.J. (2012). Self-assembling nanoparticles for intra-articular delivery of anti-inflammatory proteins. Biomaterials.

[11] Rahman M., Laurent S., Tawil N., Yahia L., Mahmoudi M. (2013). Protein-Nanoparticle Interactions.

[12] Partikel K., Korte R., Stein N.C., Mulac D., Herrmann F.C., Humpf H.-U., Langer K. (2019). Effect of nanoparticle size and PEGylation on the protein corona of PLGA nanoparticles. Eur. J. Pharm. Biopharm..

[13] Torchilin V., Levchenko T., Lukyanov A., Khaw B., Klibanov A., Rammohan R., Samokhin G., Whiteman K. (2001). p-Nitrophenylcarbonyl-PEG-PE-liposomes: Fast and simple attachment of specific ligands, including monoclonal antibodies, to distal ends of PEG chains via p-nitrophenylcarbonyl groups. Biochim. Et Biophys. Acta (BBA) Biomembr..

[14] Xia Y., Tian J., Chen X. (2016). Effect of surface properties on liposomal siRNA delivery. Biomaterials.

[15] Blume G., Cevc G., Crommelin M., Bakker-Woudenberg I., Kluft C., Storm G. (1993). Specific targeting with poly(ethylene glycol)-modified liposomes: Coupling of homing devices to the ends of the polymeric chains combines effective target binding with long circulation times. Biochim. Et Biophys. Acta (BBA) Biomembr..

[16] Li M., Jiang S., Simon J., Paßlick D., Frey M.L., Wagner M., Mailänder V., Crespy D., Landfester K. (2021). Brush Conformation of Polyethylene Glycol Determines the Stealth Effect of Nanocarriers in the Low Protein Adsorption Regime. Nano Lett..

[17] Bolourchian N., Mahboobian M.M., Dadashzadeh S. (2013). The effect of PEG molecular weights on dissolution behavior of simvastatin in solid dispersions. Iran. J. Pharm. Res..

[18] Pelaz B., del Pino P., Maffre P., Hartmann R., Gallego M., Rivera-Fernández S., de la Fuente J.M., Nienhaus G.U., Parak W.J. (2015). Surface Functionalization of Nanoparticles with Polyethylene Glycol: Effects on Protein Adsorption and Cellular Uptake. ACS Nano.

[19] Yamaoka T., Tabata Y., Ikada Y. (1994). Distribution and Tissue Uptake of Poly(ethylene glycol) with Different Molecular Weights after Intravenous Administration to Mice. J. Pharm. Sci..

[20] Owensiii D., Peppas N. (2006). Opsonization, biodistribution, and pharmacokinetics of polymeric nanoparticles. Int. J. Pharm..

[21] Gombotz W.R., Guanghui W., Horbett T.A., Hoffman A.S. (1991). Protein adsorption to poly(ethylene oxide) surfaces. J. Biomed. Mater. Res..

[22] Gref R., Lück M., Quellec P., Marchand M., Dellacherie E., Harnisch S., Blunk T., Müller R. (2000). ‘Stealth’ corona-core nanoparticles surface modified by polyethylene glycol (PEG): Influences of the corona (PEG chain length and surface density) and of the core composition on phagocytic uptake and plasma protein adsorption. Colloids Surf. B Biointerfaces.

[23] Reboredo C., González-Navarro C.J., Martínez-Oharriz C., Martínez-López A.L., Irache J.M. (2021). Preparation and evaluation of PEG-coated zein nanoparticles for oral drug delivery purposes. Int. J. Pharm..

[24] Wang Y., Wang J., Sun M., Zhang J., Bi Y. (2022). Coating liposomes with ring-like PEG: The synthesis and stealth effect of cholesterol–PEG–cholesterol. Mater. Adv..

[25] Tong S., Hou S., Ren B., Zheng Z., Bao G. (2011). Self-Assembly of Phospholipid–PEG Coating on Nanoparticles through Dual Solvent Exchange. Nano Lett..

[26] Kataoka K. (1994). Design of Nanoscopic Vehicles for Drug Targeting Based on Micellization of Amphiphiuc Block Copolymers. J. Macromol. Sci. Part. A.

[27] Gong C., Wei X., Wang X., Wang Y., Guo G., Mao Y., Luo F., Qian Z. (2010). Biodegradable self-assembled PEG–PCL–PEG micelles for hydrophobic honokiol delivery: I. Preparation and characterization. Nanotechnology.

[28] Mordechai S., Sahu R.K., Hammody Z., Mark S., Kantarovich K., Guterman H., Podshyvalov A., Goldstein J., Argov S. (2004). Possible common biomarkers from FTIR microspectroscopy of cervical cancer and melanoma. J. Microsc..

[29] Mackanos M.A., Contag C.H. (2009). FTIR microspectroscopy for improved prostate cancer diagnosis. Trends Biotechnol..

[30] Erukhimovitch V., Pavlov V., Talyshinsky M., Souprun Y., Huleihel M. (2005). FTIR microscopy as a method for identification of bacterial and fungal infections. J. Pharm. Biomed. Anal..

[31] Katsarava R., Beridze V., Arabuli N., Kharadze D., Chu C.C., Won C.Y. (1999). Amino acid-based bioanalogous polymers. Synthesis, and study of regular poly(ester amide)s based on bis(?-amino acid) ?,?-alkylene diesters, and aliphatic dicarboxylic acids. J. Polym. Sci. A Polym. Chem..

[32] Gref R., Minamitake Y., Peracchia M.T., Trubetskoy V., Torchilin V., Langer R. (1994). Biodegradable Long-Circulating Polymeric Nanospheres. Science.

[33] Li M., De P., Li H., Sumerlin B.S. (2010). Conjugation of RAFT-generated polymers to proteins by two consecutive thiol–ene reactions. Polym. Chem..

[34] Sinha A.K., Equbal D. (2019). Thiol−Ene Reaction: Synthetic Aspects and Mechanistic Studies of an Anti-Markovnikov-Selective Hydrothiolation of Olefins. Asian J. Org. Chem..

[35] Lluch C., Ronda J.C., Galià M., Lligadas G., Cádiz V. (2010). Rapid Approach to Biobased Telechelics through Two One-Pot Thiol−Ene Click Reactions. Biomacromolecules.

[36] Nair D.P., Podgórski M., Chatani S., Gong T., Xi W., Fenoli C.R., Bowman C.N. (2014). The Thiol-Michael Addition Click Reaction: A Powerful and Widely Used Tool in Materials Chemistry. Chem. Mater..

[37] Vert M., Doi Y., Hellwich K.-H., Hess M., Hodge P., Kubisa P., Rinaudo M., Schué F. (2012). Terminology for biorelated polymers and applications (IUPAC Recommendations 2012). Pure Appl. Chem..

[38] Moghimi S.M., Hedeman H., Muir I.S., Illum L., Davis S.S. (1993). An investigation of the filtration capacity and the fate of large filtered sterically-stabilized microspheres in rat spleen. Biochim. Et. Biophys. Acta (BBA) Gen. Subj..

[39] Choi H.S., Liu W., Misra P., Tanaka E., Zimmer J.P., Ipe B.I., Bawendi M.G., Frangioni J.V. (2007). Renal clearance of quantum dots. Nat. Biotechnol..

[40] Longmire M., Choyke P.L., Kobayashi H. (2008). Clearance properties of nano-sized particles and molecules as imaging agents: Considerations and caveats. Nanomedicine.

[41] Sather A.C., Berryman O.B., Rebek J. (2010). Selective recognition and extraction of the uranyl ion. J. Am. Chem. Soc..

[42] Katsarava R. (2021). Pseudo-Proteins and Related Synthetic Amino Acid-Based Polymers Promising for Constructing Artificial Vaccines. Synthetic Peptide Vaccine Models.

[43] Fessi H., Puisieux F., Devissaguet J.P., Ammoury N., Benita S. (1989). Nanocapsule formation by interfacial polymer deposition following solvent displacement. Int. J. Pharm..

[44] Kantaria T., Kantaria T., Kobauri S., Ksovreli M., Kachlishvili T., Kulikova N., Tugushi D., Katsarava R. (2016). Biodegradable Nanoparticles Made of Amino-Acid-Based Ester Polymers: Preparation, Characterization, and In Vitro Biocompatibility Study. Appl. Sci..

[45] Zavradashvili N., Sarisozen C., Titvinidze G., Otinashvili G., Kantaria T., Tugushi D., Puiggali J., Torchilin V.P., Katsarava R. (2019). Library of Cationic Polymers Composed of Polyamines and Arginine as Gene Transfection Agents. ACS Omega.

