# Supplementary material for: PEGylated Micro/Nanoparticles Based on Biodegradable Poly(Ester Amides): Preparation and Study of the Core–Shell Structure by Synchrotron Radiation-Based FTIR Microspectroscopy and Electron Microscopy
Source: Int J Mol Sci. 2024 Jun 26;25(13):6999. doi: 10.3390/ijms25136999 (PMC11241343; doi:10.3390/ijms25136999)
Supplement: Supplementary file 1 [file ijms-25-06999-s001.zip › ijms-3054014-supplementary.pdf]

PEGylated Micro/Nanoparticles Based on Biodegradable Poly(Ester Amides): Preparation and Study of the Core-Shell Structure by Synchrotron-Radiation Based FTIR Microspectroscopy and Electron Microscopy

Supplementary Information

Davit Makharadze [1], Temur Kantaria [2], Ibraheem Yousef [3], Luis del Valle [1,4], Ramaz Katsarava [2] and Jordi Puiggalí [1,4,*]

1 Departament de Enginyeria Química, Universitat Politècnica de Catalunya, EEBE, Av. Eduard Maristany 10-14, E-08019 Barcelona, Spain; davit.makharadze@upc.edu (D.M.); luis.javier.del.valle@upc.edu (L.J.)
2 Institute of Chemistry and Molecular Engineering, Agricultural University of Georgia, Tbilisi, 0159, Georgia; t.kantaria@agruni.edu.ge (T.K.) , r.katsarava@agruni.edu.ge (R.K.)
3 ALBA Synchrotron Light Facility, Carrer de la Llum 2-26, Cerdanyola del Vallès, 08290 Barcelona, Spain; iyousef@cells.es (I.Y.)
4 Barcelona Research Center in Multiscale Science and Engineering, Universitat Politècnica de Catalunya, Campus Diagonal-Besòs, Av. Eduard Maristany 10-14, E-08019 Barcelona, Spain.
* Correspondence: jordi.puiggali@upc.edu (J.P.)

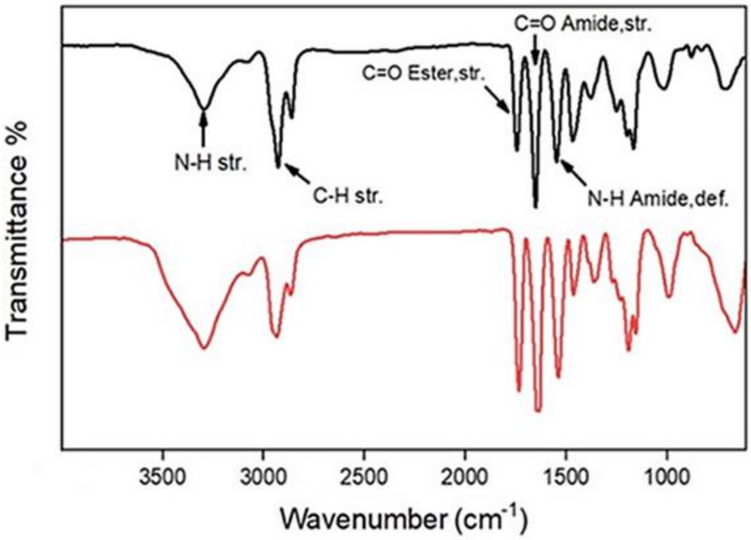

Figure S1. FTIR spectra of saturated poly(esteramide) 8L6 (black) and unsaturated co-poly(esteramide) $(FuL6)_{0.5}$-$(8L6)_{0.5}$ (red).

Characteristic FTIR absorption bands of ester C=O stretching at 1736 cm^{-1}, amide (amides A, B, I, and II at 3300, 3080, 1635, and 1538 cm^{-1}, respectively), and aliphatic (asymmetric and symmetric CH_2 stretching and CH_3 stretching at 2917, 2850 and 2955 cm^{-1}, respectively) groups were detected.

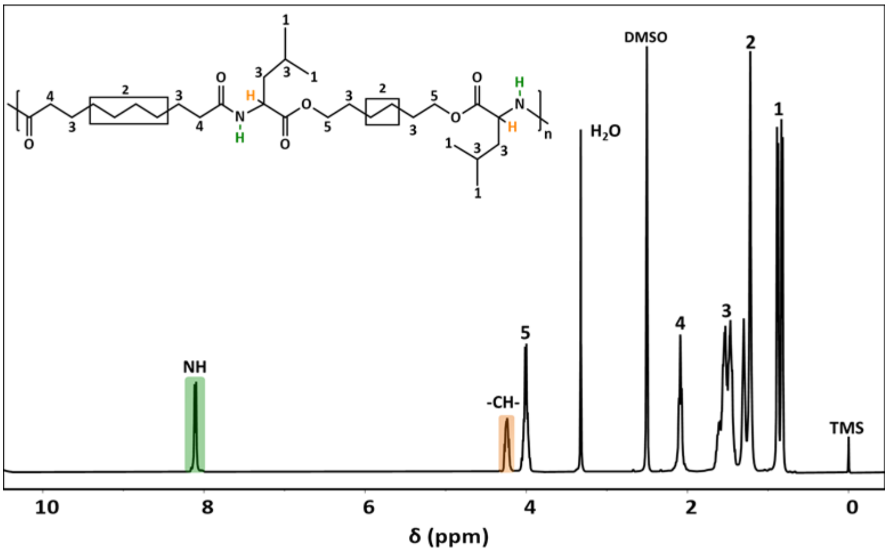

Figure S2. ^{1}H-NMR spectrum of polymer 8L6.

^{1}H-NMR spectrum of the 8L6 polymer: 0.84-0.98 (12H, $-CH_3$), 1.18-1.33 (12H, $O-(CH_2)_2-(C\underline{H}_2)_2-(CH_2)_2-O-$ and $-(O)C-(CH_2)_2-(C\underline{H}_2)_4-(CH_2)_2-C(O)-$), 1.34-1.80 (14H, $-C\underline{H}(CH_3)_2$, $C\underline{H}_2-CH(CH_3)_2$, $-O-CH_2-C\underline{H}_2$ and $-(O)C-CH_2-C\underline{H}_2$), 2.16-2.41 (4H, $-(O)C-CH_2$), 4.15 (4H, $O-CH_2$), 4.66 (2H, $NH-C\underline{H}$) and 5.99-6.2 (2H, NH).

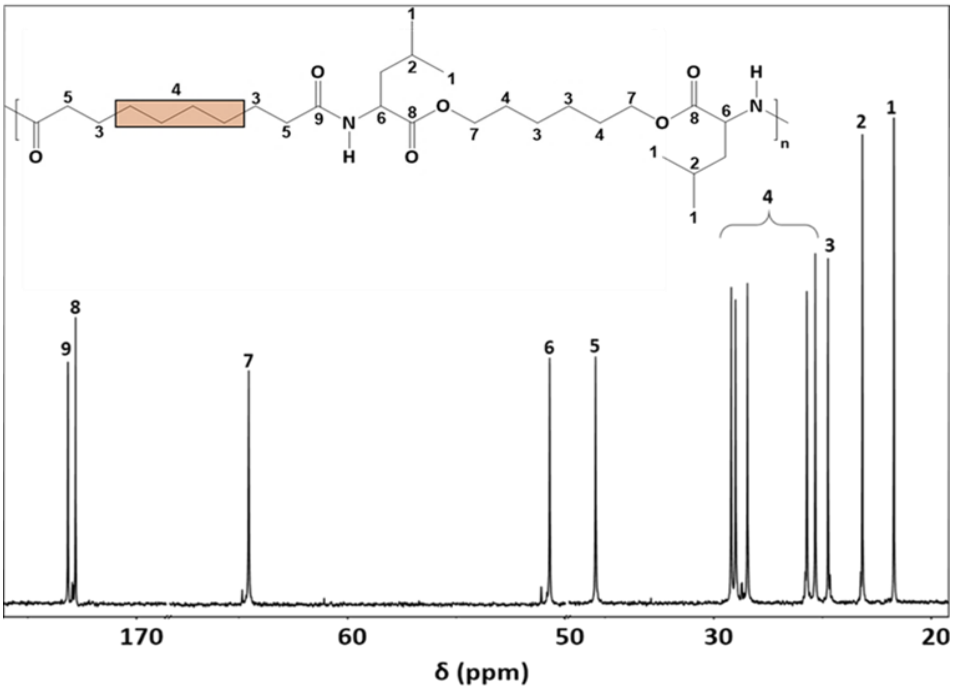

Figure S3. ^{13}C-NMR spectrum of polymer 8L6.

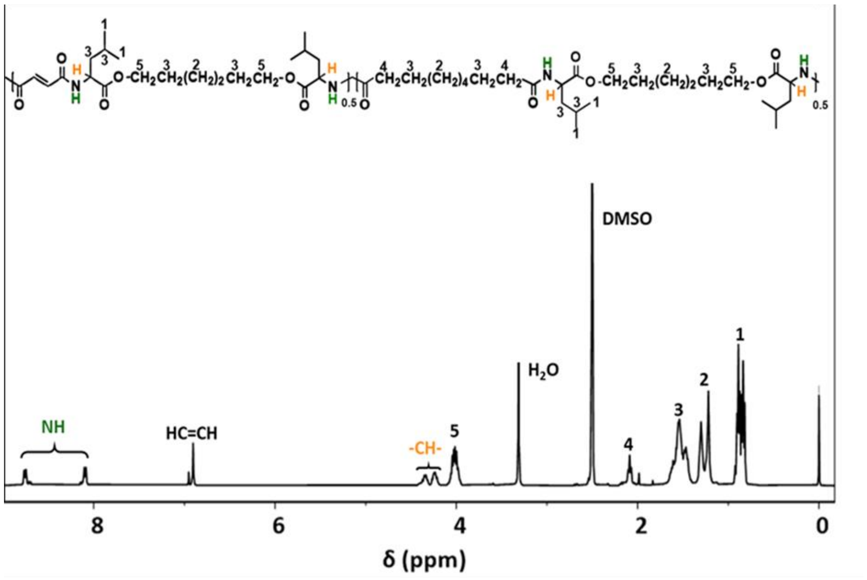

Figure S4. ^{1}H-NMR spectrum of unsaturated co-poly(ester amide) $(FuL6)_{0.5}$-$(8L6)_{0.5}$.

The ^{1}H-NMR spectrum of the copolymer showed signals at 0.83-0.91 (12H, $-CH_3$), 1.23-1.31 (8H,$-O-(CH_2)_2-(C\underline{H_2})_2-(CH_2)_2-O-$ and $-(O)C-(CH_2)_2-(C\underline{H_2})_4-(CH_2)_2-C(O)-$, stack), 1.48-1.62 (12H, $-C\underline{H}(CH_3)_2$; $C\underline{H_2}-CH(CH_3)_2$; $-O-CH_2-C\underline{H_2}$ and $-(O)C-CH_2-C\underline{H_2}$, stack), 2.10 (2H, $-(O)C-CH_2$), 4.02 (4H, $O-CH_2$), 4.25-4.35 (2H, $NH-C\underline{H}$), 6.91 (1H, -CH=CH), 8.10 and 8.77 (2H, NH)

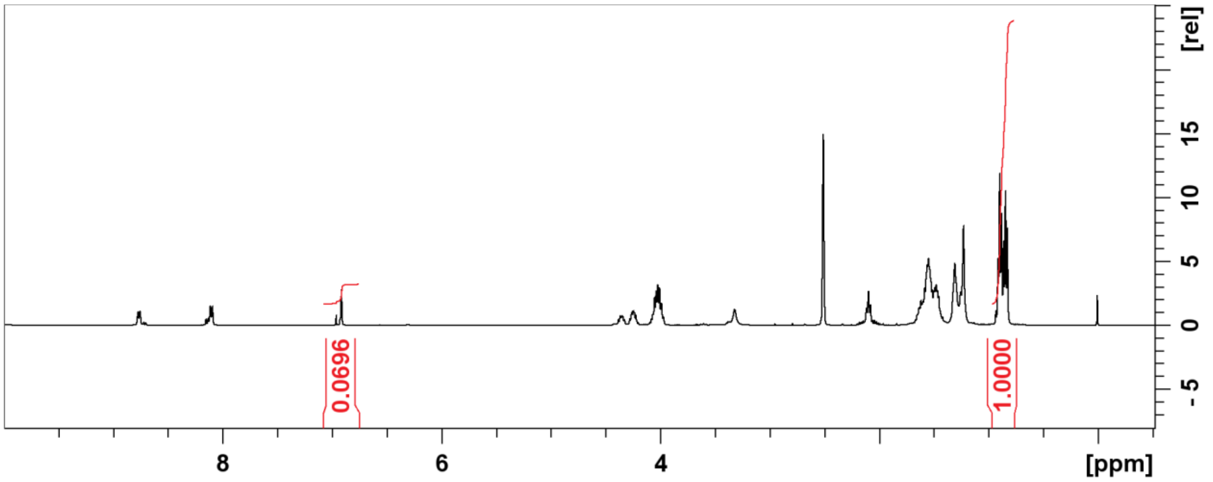

Figure S5. Integration results of unsaturated co-poly(esteramide) $(FuL6)_{0.5}$-$(8L6)_{0.5}$.

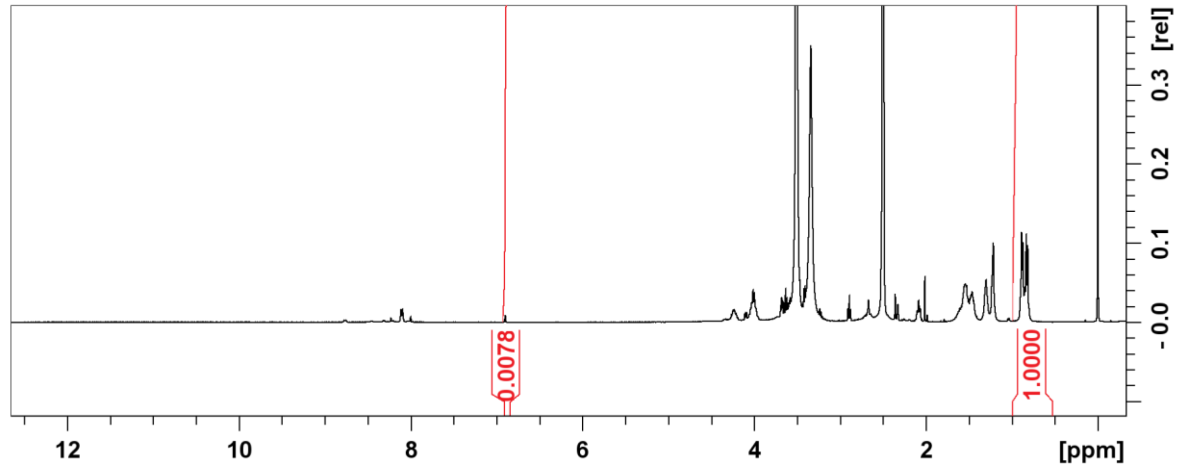

Figure S6. Integration results of PEG-PEA surfactant.
